# Supplementary material for: Fairy circles in Namibia are assembled from genetically distinct grasses
Source: Commun Biol. 2020 Nov 20;3:698. doi: 10.1038/s42003-020-01431-0 (PMC7680098; doi:10.1038/s42003-020-01431-0)
Supplement: Supplementary file 1 — Supplementary Information [file 42003_2020_1431_MOESM1_ESM.pdf]

SUPPLEMENTARY INFORMATION

SUPPLEMENTARY FIGURES

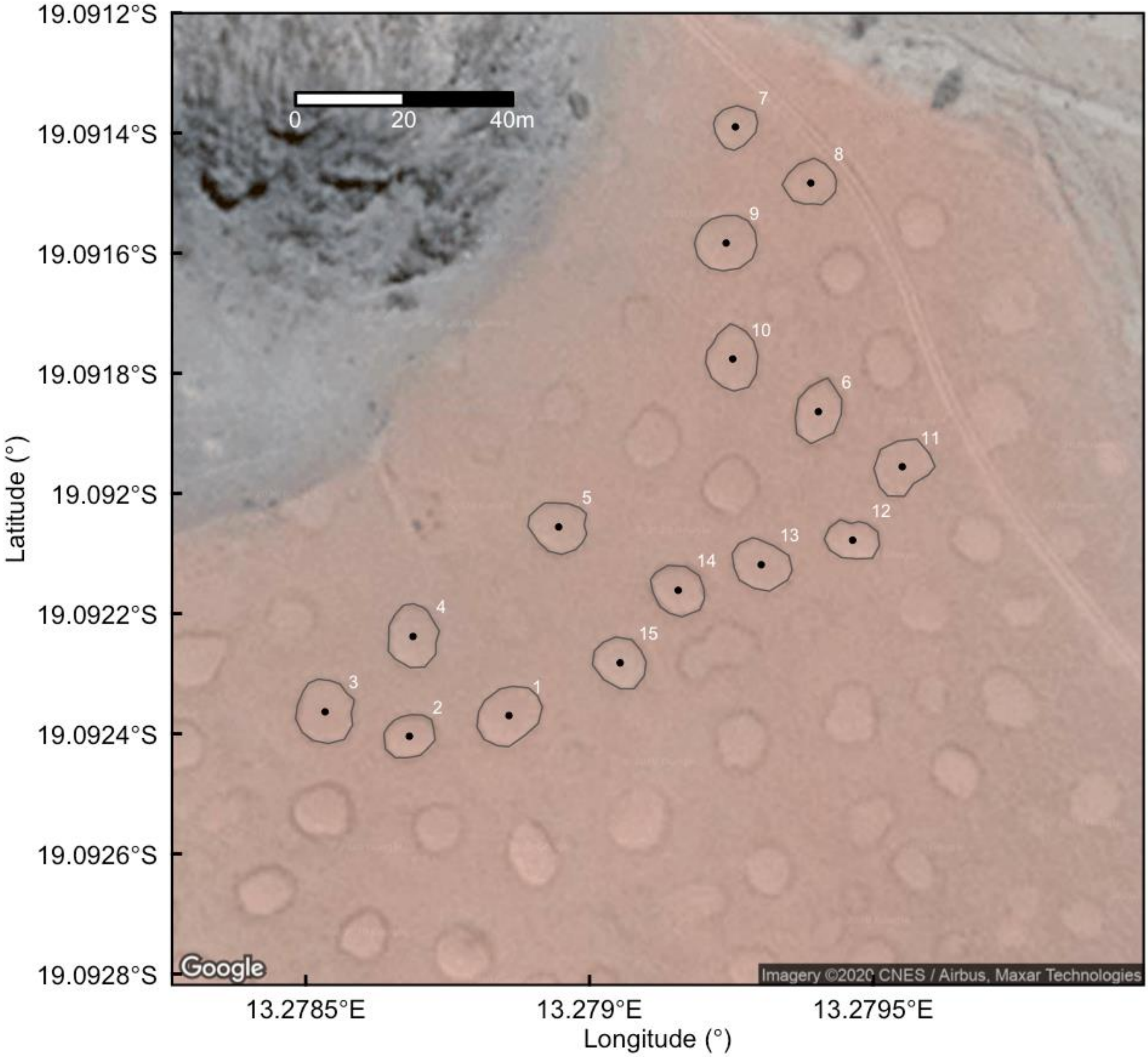

Supplementary Figure 1: The *S. uniplumis* sampling site

The 15 FCs of *S. uniplumis* that were sampled are indicated.

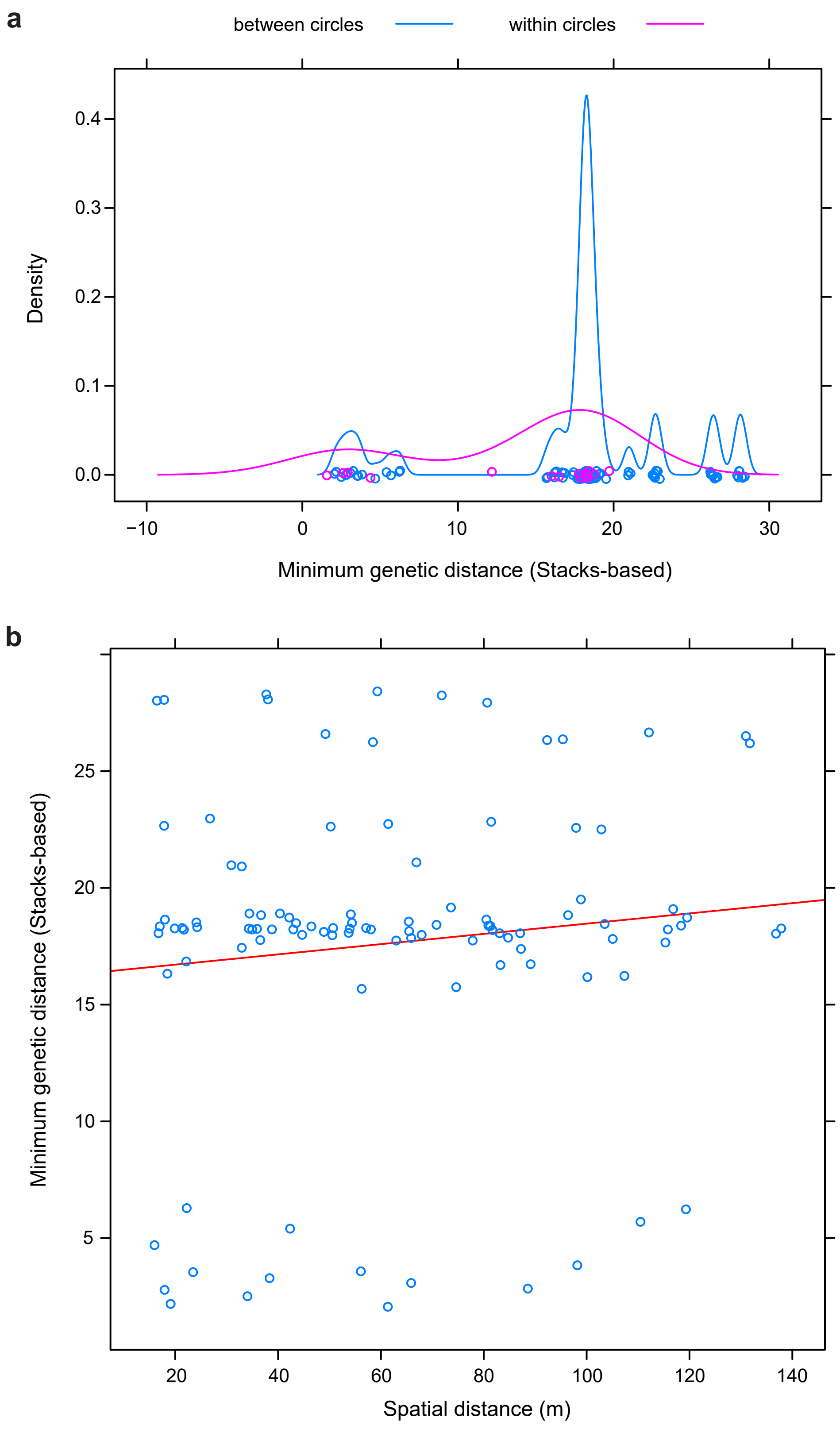

a
between circles
within circles
Density
0.4
0.3
0.2
0.1
0.0
−10
0
10
20
30
Minimum genetic distance (Stacks-based)
b
Minimum genetic distance (Stacks-based)
25
20
15
10
5
20
40
60
80
100
120
140
Spatial distance (m)

Supplementary Figure 2: Relationship of within- and between circle genetic distances based on Stacks

(a) Density distributions of minimal genetic distances between samples from within the 15 *S. uniplumis* circles (purple; n=15 within-circle distances) and between all possible pairs of circles (blue; n=105 between-circle distances) are shown. Small circles indicate individual distances.

(b) For each pair of *S. uniplumis* FCs the minimal genetic distance between samples from the two FCs are plotted relative to the spatial distance between the FCs. No within-circle comparisons are shown. The regression line is shown in red.

Stacks-based

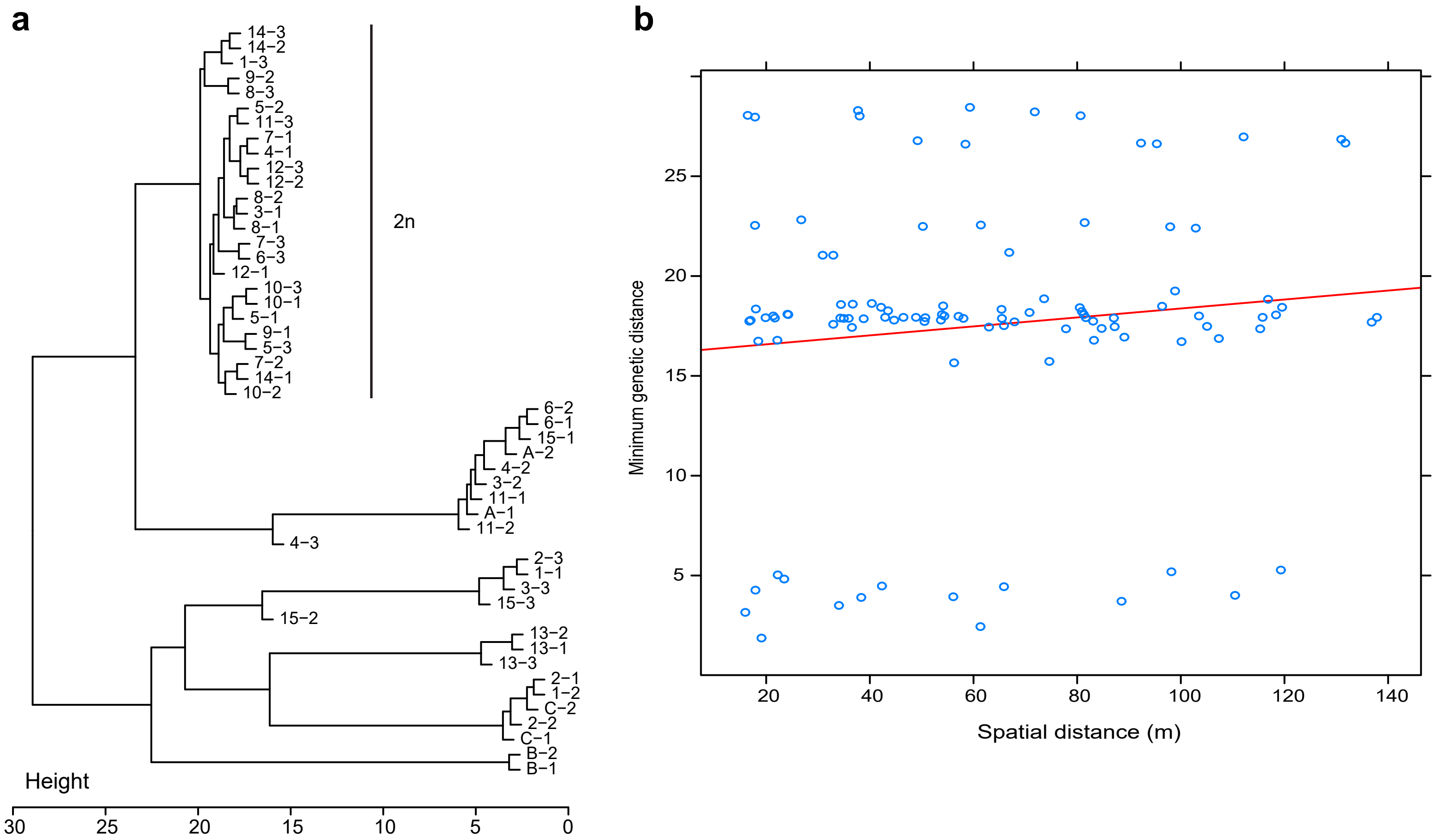

O. sativa mapping-based

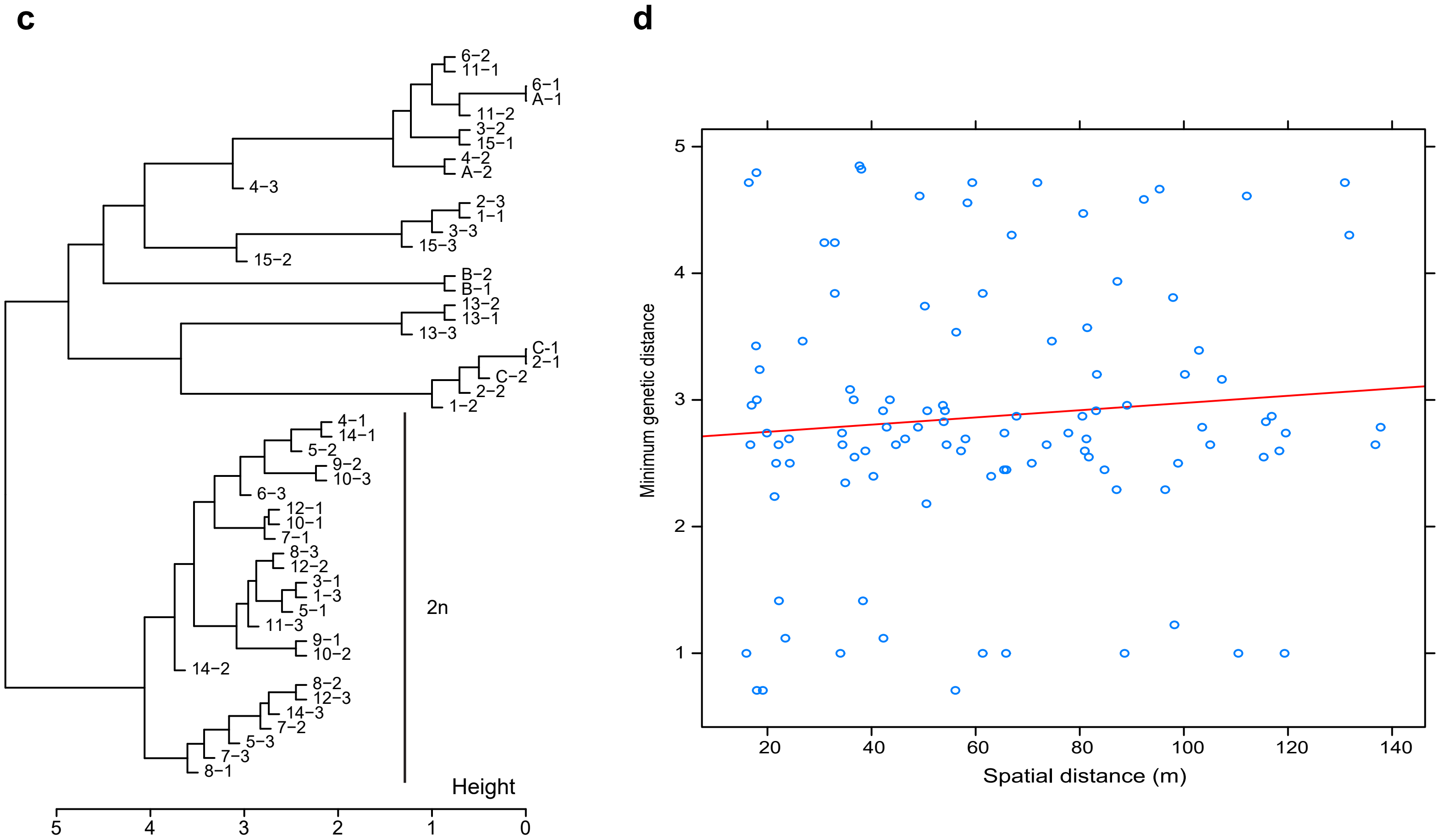

Supplementary Figure 3: Sensitivity of the analyses to parameters and allele frequencies

(a,c) Hierarchical clustering of *S. uniplumis* samples across 15 circles, as in Figure 2a, except that genotypes were recoded with a 10% alternative-allele frequency threshold for heterozygotes and only variants were considered that were found in at least two individuals. Data in (a) are based on the stacks analysis, in (c) on the mapping to the *O. sativa* genome, and read depth of 11 reads in each sample was required. Diploid samples are indicated (2n), the ones above are tetraploid, except for the octaploid sample 4-3.

(b,d) For each pair of FCs the minimal genetic distance between samples from the two FCs are plotted relative to the spatial distance between the FCs. No within-circle comparisons are shown. The regression line is shown in red. Genetic distances in (b) are based on the analysis shown in (a), genetic distances in (d) on the analysis shown in (c).
