# Supplementary material for: Fairy circles in Namibia are assembled from genetically distinct grasses
Source: Commun Biol. 2020 Nov 20;3:698. doi: 10.1038/s42003-020-01431-0 (PMC7680098; doi:10.1038/s42003-020-01431-0)
Supplement: Supplementary file 3 — Description of Additional Supplementary Files [file 42003_2020_1431_MOESM3_ESM.pdf]

Description of Additional Supplementary Files

File Name: Supplementary Data 1

Description: Raw data used for figures 2a/2b and Figures 3a/3c.
