# Supplementary material for: Fairy circles in Namibia are assembled from genetically distinct grasses
Source: Commun Biol. 2020 Nov 20;3:698. doi: 10.1038/s42003-020-01431-0 (PMC7680098; doi:10.1038/s42003-020-01431-0)
Supplement: Supplementary file 4 — Reporting Summary [file 42003_2020_1431_MOESM4_ESM.pdf]

nature research

Corresponding author(s): Jeremy Midgley; Michael Lenhard

Last updated by author(s): Oct 1, 2020

Reporting Summary

Nature Research wishes to improve the reproducibility of the work that we publish. This form provides structure for consistency and transparency in reporting. For further information on Nature Research policies, see our Editorial Policies and the Editorial Policy Checklist.

Statistics

For all statistical analyses, confirm that the following items are present in the figure legend, table legend, main text, or Methods section.

n/a	Confirmed	
☐	☒	The exact sample size (*n*) for each experimental group/condition, given as a discrete number and unit of measurement
☐	☒	A statement on whether measurements were taken from distinct samples or whether the same sample was measured repeatedly
☒	☐	The statistical test(s) used AND whether they are one- or two-sided<br>*Only common tests should be described solely by name; describe more complex techniques in the Methods section.*
☒	☐	A description of all covariates tested
☒	☐	A description of any assumptions or corrections, such as tests of normality and adjustment for multiple comparisons
☒	☐	A full description of the statistical parameters including central tendency (e.g. means) or other basic estimates (e.g. regression coefficient) AND variation (e.g. standard deviation) or associated estimates of uncertainty (e.g. confidence intervals)
☒	☐	For null hypothesis testing, the test statistic (e.g. *F*, *t*, *r*) with confidence intervals, effect sizes, degrees of freedom and *P* value noted<br>*Give P values as exact values whenever suitable.*
☒	☐	For Bayesian analysis, information on the choice of priors and Markov chain Monte Carlo settings
☒	☐	For hierarchical and complex designs, identification of the appropriate level for tests and full reporting of outcomes
☒	☐	Estimates of effect sizes (e.g. Cohen's *d*, Pearson's *r*), indicating how they were calculated

Our web collection on statistics for biologists contains articles on many of the points above.

Software and code

Policy information about availability of computer code

Data collection	no software was used to collect data
Data analysis	stacks version 2.41; bwa mem; bwsamtools; bcftools; vcftools; R

For manuscripts utilizing custom algorithms or software that are central to the research but not yet described in published literature, software must be made available to editors and reviewers. We strongly encourage code deposition in a community repository (e.g. GitHub). See the Nature Research guidelines for submitting code & software for further information.

Data

Policy information about availability of data

All manuscripts must include a data availability statement. This statement should provide the following information, where applicable:

- Accession codes, unique identifiers, or web links for publicly available datasets
- A list of figures that have associated raw data
- A description of any restrictions on data availability

ddRAD sequencing data are available at NCBI SRA under accession number PRJNA576806

Field-specific reporting

Please select the one below that is the best fit for your research. If you are not sure, read the appropriate sections before making your selection.

☐ Life sciences ☐ Behavioural & social sciences ☒ Ecological, evolutionary & environmental sciences

For a reference copy of the document with all sections, see nature.com/documents/nr-reporting-summary-flat.pdf

Ecological, evolutionary & environmental sciences study design

All studies must disclose on these points even when the disclosure is negative.

Study description	Not applicable
Research sample	Leaf samples were taken from adult viable plants of two species of Stipagrostis grass, S. uniplumis and S. ciliata from two different study sites. These grasses dominate Fairy Circles.
Sampling strategy	For S. ciliata and S. uniplumis we sampled five and fifteen circles respectively, collecting leaf material from three individual plants per circle. Two of the samples ("1" and "2") were located within 2m of each other, while the third ("3") was chosen on the opposite side of the circle. For S. uniplumis, we also took two samples, each from one tuft in three unrelated circles ("A", "B" and C") to test for genetic relatedness within a tuft. Testing whether two plants in one Fairy Circle are not identical would disprove the clonality hypothesis. Nevertheless, we sampled three plants per circle, for five and fifteen circles, for two species of grass.
Data collection	JJM, MC and NB collected field samples. ML, CK, CNH and NI extracted and analyzed the data from the samples.
Timing and spatial scale	Samples of S. ciliata were collected in 2017, and S. uniplumis in 2018.
Data exclusions	None
Reproducibility	We did not repeat the experiment.
Randomization	We did not randomize the samples.
Blinding	Not relevant.

Did the study involve field work? ☒ Yes ☐ No

Field work, collection and transport

Field conditions	Not applicable
Location	S. uniplumis (15 circles) and S. ciliata (5 circles) perimeter grasses were sampled at 19o05′31.58″ S, 13o16′44.54″ E and 24 o 56′ 32.87″ S, 16 o 01′ 12.82″ E sites respectively .
Access & import/export	Samples were collected under Permit 1854 (Ministry of Environment and Tourism, Namibia) and Permit NRNR/P/2014/01 (NamibRand Nature Reserve)
Disturbance	Disturbance was minimal. Only two leaves were removed per tussock of grass.

Reporting for specific materials, systems and methods

We require information from authors about some types of materials, experimental systems and methods used in many studies. Here, indicate whether each material, system or method listed is relevant to your study. If you are not sure if a list item applies to your research, read the appropriate section before selecting a response.

Materials & experimental systems

n/a	Involved in the study	
☒	☐	Antibodies
☒	☐	Eukaryotic cell lines
☒	☐	Palaeontology and archaeology
☒	☐	Animals and other organisms
☒	☐	Human research participants
☒	☐	Clinical data
☒	☐	Dual use research of concern

Methods

n/a	Involved in the study	
☒	☐	ChIP-seq
☒	☐	Flow cytometry
☒	☐	MRI-based neuroimaging
